# Interoceptive Awareness and Female Orgasm Frequency and Satisfaction

**DOI:** 10.3390/brainsci14121236

**Published:** 2024-12-09

**Authors:** Emily Dixon, Giulia L. Poerio, Gerulf Rieger, Megan Klabunde

**Affiliations:** 1 Department of Psychology, University of Essex, Wivenhoe Park, Colchester CO4 3SQ, UK; 2 School of Psychology, University of Sussex, Falmer, Brighton BN1 9RH, UK

**Keywords:** interoception, orgasm, orgasm frequency, orgasm satisfaction, women, orgasm gap

## Abstract

Background: The female orgasm is a highly understudied phenomenon that is linked to both wellbeing and relationship satisfaction in women. Although orgasm has been associated with interoception—the sense of the physiological condition of the body—very few studies have directly examined the influence that interoception has on orgasm. Objectives: This study investigates how the subjective experience of one’s interoceptive capacities (called interoceptive awareness) is associated with self-reported orgasm frequency and satisfaction in people who identify as women. Methods: In a dataset of 318 women, orgasm frequency and satisfaction were both rated significantly higher for solitary as compared to partnered sexual experiences. Results: Analysis of how dimensions of interoceptive awareness correlated with orgasm frequency and satisfaction showed that (1) ‘Noticing’ predicted orgasm frequency (but not satisfaction) across both solitary and partnered interactions, (2) ‘Attention Regulation’ predicted greater frequency and satisfaction of solitary orgasm (but not partnered interactions), and (3) ‘Body Trusting’ predicted orgasm satisfaction (but not frequency) across both solitary and partnered contexts. Conclusions: Findings underscore the importance of moving beyond orgasmic dysfunction research by investigating how interoception is associated with healthy—and potentially even optimal—orgasmic functioning in women.

## 1. Introduction

The human orgasm is possibly the most pleasurable experience men and women can naturally experience, releasing large quantities of feel-good hormones such as dopamine, oxytocin, and serotonin. It is theorised to include four main physiological stages that lead towards a sexual climax in women, such as (1) an excitement phase where the body initially prepares for sexual intercourse, (2) a plateau phase where one’s heart rate increases in response to sexual pleasure and stimulation, (3) an orgasmic phase whereby vaginal and uterine muscles contract and a woman subjectively reports having an orgasm, and (4) it finishes with a resolution phase where one’s muscles return to their relaxed state [1]. The female orgasm has been a topic of much controversy, as it has been previously thought that orgasms are not relevant for sexual functioning in women. Therefore, very few studies have actually examined the orgasm and its theoretical models in women. This is problematic, however, as recent studies have demonstrated that the female orgasm does—in fact—play a vital role in sexual functioning for women. It is linked to increased sexual pleasure, satisfaction, and sexual desire and also contributes to both mate selection and pair bonding for women [2]. Orgasms are also important for the overall wellbeing of women, contributing positively to greater relationship wellbeing and satisfaction, life satisfaction, and psychological and physical functioning [3,4,5,6,7]. Despite the many benefits of orgasms for women, to date, research has tended to focus on orgasmic *dysfunction*, with a paucity of evidence underlying normal—and even less so for optimal—orgasmic functioning.

Interoception—the sense of the internal condition of the body—has been conceptualised as a sensory modality similar to other sensations arising from the viscera, such as hunger, nausea, and the sense of one’s heartbeat and breathing [8]. The late neuroanatomist Bud Craig specifically categorised orgasm as an interoceptive sense, given that its sensory tract organisation follows similar pathways to the brain as other senses that are also classified as interoceptive [8]. This interoceptive pathway includes small-diameter afferent fibres (C-fibres or CT afferents) that are associated with the transmission of interoceptive sensations. They represent the physiological condition of the body through the spinothalamic-cortical system, a homeostatic afferent pathway that arises from the viscera and periphery of the body and projects to similar parts of the brain. Interoception functions in accordance with our homeostatic needs; heat is pleasant when we are too cold, and cool water is pleasant when we are too hot [8]. The interoceptive system is distinct from senses that are considered exteroceptive in that while the exteroceptive system relates to external stimuli and projects to the somatosensory cortex, the interoceptive system is represented in the insula, which is considered as “the interoceptive cortex” [9].

Biologically, there are several ways that link interoceptive processing to orgasm: the first being an anatomical sensory and touch-related link, the second a cognitive and affective link, and the third via the vagus nerve. Firstly, unmyelinated sensory C-fibres are activated by “emotional touch experiences”, which involve slowly stroking the hairy part of the skin. They contrast with the discriminative touch functions of A-fibres that typically exist on the non-hairy parts of the skin (i.e., such as the hands [10]). Largely, C-fibres—including those involved in emotional touch—target the insular cortex of the brain [11,12]. However, previous research has only found C-fibres in the hairy skin of humans and not the glabrous skin such as the genitalia. Cazala, Vienney, and Stoléru [13] note that the skin of the external female genitalia is packed with touch receptors, classified as free nerve endings associated with hair follicles—a description bearing many similarities with C-fibres. Free nerve endings are the terminals of small-diameter C and Aẟ-fibres, which have been found in the glabrous skin of mammals [14]. Thus, sexual stimulation of female genitalia projects to both the somatosensory cortex and the posterior insula. The more physical, sensory aspects of orgasm and touch (through Aβ afferents) activate the somatosensory cortices, while the affective aspects of orgasm and touch (through C-fibres) are suggested to be more associated with activation of the posterior insula [13,15].

Second, in addition to being activated through C-fibres, the anterior insula has connections to the prefrontal cortex and is associated with one’s metacognitive representation of the internal condition of the body [16]. The anterior insula is also activated through visual sexual stimuli [10]. Thus, much like in the way that interoception has been said to represent both physiological senses and their related affective components [8], orgasm has been recognised to include both sensory and affective dimensions [17].

Lastly, interoception and orgasm are also linked via the vagus nerve. Recent studies indicate that 80% of the vagus nerve can be classified as interoceptive, as the vagus nerve largely consists of c-fibre afferents that follow the spinal-thalamic afferent pathway to the brain [18]. In addition to various other internal organs within the body, the vagus nerve also innervates the cervix and the uterus, which are associated with orgasmic stimulation and its associated uterine contractions, respectively [19,20]. Cervical stimulation during sexual interactions also impacts similar parts of the brain as vaginal and clitoral stimulation, further indicating the cervix’s role in sexual functioning and its link to interoception [21].

Despite the clear biological, anatomical, and neural connections linking interoception to orgasm, little is known about the relationship between interoception and the subjective experience of orgasm in women. A few studies, however, by Handy and Meston, examined the link between interoceptive awareness and *sexual arousal* in women. Specifically, they looked at sexual concordance—the extent to which an agreement between one’s perceived and actual physiological sexual arousal occurs—and found that Body Listening on the self-report Multidimensional Assessment of Interoceptive Awareness (MAIA) scale is positively associated with subjective ratings of genital arousal and also that Noticing on the MAIA is positively associated with higher sexual concordance [22]. The authors replicated the first finding linking Body Listening to genital arousal in 26 women with female sexual arousal disorder (FSAD). A link between Noticing and sexual concordance was not found in those with FSAD [23]. A third study by Handy and Meston directly compared 34 women with and 33 women without sexual arousal disturbance and found that interoceptive awareness moderated sexual concordance; individual differences, however, were observed across the sample. In women without sexual arousal disturbance, the Not Distracting and Not Worrying subscales of the MAIA were positively associated with sexual concordance. However, this finding did not apply to women with sexual arousal disturbances [24].

Our study expands these findings, which have found a link between interoceptive awareness and sexual arousal in women by (1) directly looking at interoception and orgasm, (2) examining this link for both orgasm frequency and satisfaction, and (3) comparing these links across solitary and partnered sexual interactions. Previously, we noted that the female orgasm is associated with numerous benefits for women and the larger society; these differ for orgasm frequency and satisfaction. Orgasm frequency is associated with relationship satisfaction, plays a role in long-term mate selection for women, and is also associated with a woman’s sexual assertiveness [25], whereas orgasm satisfaction is associated with a woman’s familiarity with one’s partner [26]. It is unknown, however, how orgasm frequency and satisfaction are impacted by interoceptive awareness in women. This is critical to identify, as it may elucidate separate and distinct mechanisms that—for the first time—indicate how a woman can specifically enhance either her orgasm frequency or satisfaction (or both!). Additionally, rather than continuing to focus on female sexual dysfunction, we anticipate that the identification of interoceptive-specific mechanisms will facilitate the development of targeted and precise body-based perceptual strategies that provide sexual enrichment in women. We have also examined these links separately for solo as compared to partnered sexual interactions in women, as they vary in measurements of both frequency and satisfaction. Overall, we predicted that (1) overall interoceptive awareness as measured by the MAIA is associated with both female orgasm frequency and satisfaction and also that (2) orgasm frequency and satisfaction will be uniquely linked to various MAIA subscales.

## 2. Materials and Methods

### 2.1. Participants

A total of 641 participants originally took part in the study, recruited primarily through volunteer social media pages; the highest number of responses were from feminist social media groups. People self-identifying as women were invited to complete an online survey and were not reimbursed for their time. Incomplete survey data (321 records) were eliminated, as well as data from individuals not meeting the participation criteria of self-identifying as a woman. A complete dataset of 360 participants was analysed for the purpose of this study using SPSS version 29 (M_age_ = 29.56, SD = 10.29, Range = 18–69). Of the 360 total participants, 333 completed the frequency section of the Female Orgasm Scale, and 318 completed the satisfaction section. Some participants provided partial responses, which accounts for the differences in completion rates across these sections. The majority (94.7%) were cis-gender women, 2 were trans men (0.6%), and 17 (4.7%) identified as non-binary with female sex at birth. The Kinsey Scale [27] was used to determine the sexual orientation of participants, using a 0–6 Likert scale, with zero indicating entirely heterosexual, six indicating entirely homosexual, and scores in between indicating degrees of bisexuality. A total of 79 participants indicated zero, 75 indicated one, 73 indicated two, 80 indicated three, 21 indicated four, 17 indicated five, and 8 indicated six. Another 7 participants indicated they did not feel sexual attraction (see Table 1).

### 2.2. Measures

Interoception. The Multidimensional Assessment of Interoceptive Awareness Scale (MAIA-2; [28]) was used to measure multiple dimensions of interoceptive awareness accessible to self-report. Participants rated each item from 0 (Never) to 5 (Always). Eight subscales provide a measure of the following dimensions of interoceptive awareness:

1. Noticing (4 items, α = 0.70), measuring the awareness of uncomfortable, comfortable, and neutral body sensations (e.g., “When I am tense, I notice where the tension is located in my body”);
2. Not-Distracting (6 items, α = 0.89), measuring the tendency not to ignore or distract oneself from sensations of pain or discomfort (e.g., “I distract myself from sensations of discomfort”—reverse-scored);
3. Not-Worrying (5 items, α = 0.78), measuring the tendency not to worry or experience emotional distress with sensations of pain or discomfort (e.g., “I can notice an unpleasant body sensation without worrying about it”);
4. Attention Regulation (7 items, α = 0.86), measuring the ability to sustain and control attention to body sensations (e.g., “I can maintain awareness of my inner bodily sensations even when there is a lot going on around me”);
5. Emotional Awareness (5 items, α = 0.81), measuring awareness of the connection between body sensations and emotional states (e.g., “I notice how my body changes when I feel happy/joyful”);
6. Self-Regulation (4 items, α = 0.82), measuring the ability to regulate distress by attention to body sensations (e.g., “I can use my breath to reduce tension”);
7. Body Listening (3 items, α = 0.83), measuring active listening to the body for insight (e.g., “I listen to my body to inform me about what to do”);
8. Trusting (3 items, α = 0.90), measuring the experience of one’s body as safe and trustworthy (e.g., “I trust my body sensations”).

Negatively worded items were reverse-coded, and items were averaged to provide scores for each subscale and an overall score where higher scores indicated greater interoceptive awareness across all dimensions measured (α = 0.89).

Orgasm frequency and satisfaction (solitary and partner). The Female Orgasm Scale (FOS; [29]) was used to measure the frequency and satisfaction with both solitary and partner orgasms. We slightly modified the measure to accommodate solitary sexual encounters in addition to partnered interactions. While the original FOS asks how often oral stimulation leads to orgasm, we adapted this question to suit solitary experiences by asking how often the use of sex toys leads to orgasm. Other questions were reworded to ask specifically about solitary experiences rather than partnered ones (see Table 2). Orgasm frequency was assessed with five items; participants rated the percentage of time (0–100%) that they achieved orgasm through various means (e.g., with a partner—during vaginal intercourse, with oral stimulation; solitary—with sex toys, with hand/manual stimulation). If the statement did not apply to them, they could select not applicable. Average scores were calculated separately for partner orgasm frequency (5 items; α = 0.71) and solitary orgasm frequency (5 items; α = 0.71), with higher scores indicating the greater frequency of orgasm achieved through the different methods described (expressed as a percentage of the time).

Orgasm satisfaction was assessed with two items. Participants rated their satisfaction with the number and quality of orgasms from 1 (very unsatisfied) to 7 (very satisfied) for both their partner and solitary orgasm experiences. Average scores were calculated separately for partner satisfaction (2 items; α = 0.92) and solitary orgasm satisfaction (5 items; α = 0.86), with higher scores indicating greater satisfaction with the number and quality of orgasm experiences.

### 2.3. Procedure

Participants were invited to complete the survey using Qualtrics online survey software through a link posted on social media sites. This study, which was described as an exploration of links between orgasm and the physiological senses of the body, comprised a short section asking for participants’ demographic information, followed by the measures described above (in that order) to be tainted by any influence of the adapted FOS Solitary. This study was approved by the University of Essex’s Psychology (ETH2021-1220, 28 September 2021) Ethics board, and all participants consented prior to participating in the study.

#### Analytical Approach

Descriptive statistics were obtained in order to characterise our participant sample (see Table 2). Age and sexual orientation were not the primary focus of this research, and since neither variable significantly correlated with any of our other measures (see Table 3), we did not control for them in subsequent analyses. Our hypotheses were examined by (1) correlations that measured the relationship between interoception and orgasm consistency and satisfaction for both partnered and solitary orgasms, as measured by the female orgasm scale (multicollinearity was not detected as the correlations between variables were not higher than 0.6) and (2) multiple regressions where all subscales of the MAIA were included in order to predict each of the outcomes.

## 3. Results

Means, standard deviations and Pearson correlations between key variables are displayed in Table 4.

### 3.1. Orgasm Frequency and Satisfaction Between Solitary and Partnered Activities

A comparison of orgasm frequency and satisfaction between partnered and solitary contexts was conducted using paired samples *t*-tests. In our sample, only 17 participants (5.3%) endorsed a 0% frequency for achieving orgasm, suggesting that the majority of our participants have previously experienced orgasm. On average, orgasm was achieved significantly more frequently in solitary (*M* = 65.46, *SD* = 24.56) compared to partner contexts (*M* = 44.49, *SD* = 25.55), *t*(332) = 13.29, *p* < 0.001, *d* = 0.52. Orgasm satisfaction was also significantly higher on average in solitary (*M* = 6.01, *SD* = 1.36) compared to partner contexts (*M* = 4.84, *SD* = 2.06), *t*(318) = 9.51, *p* < 0.001, *d* = 0.75.

### 3.2. Orgasm Frequency (Solitary and Partner)

**Figure 1:**
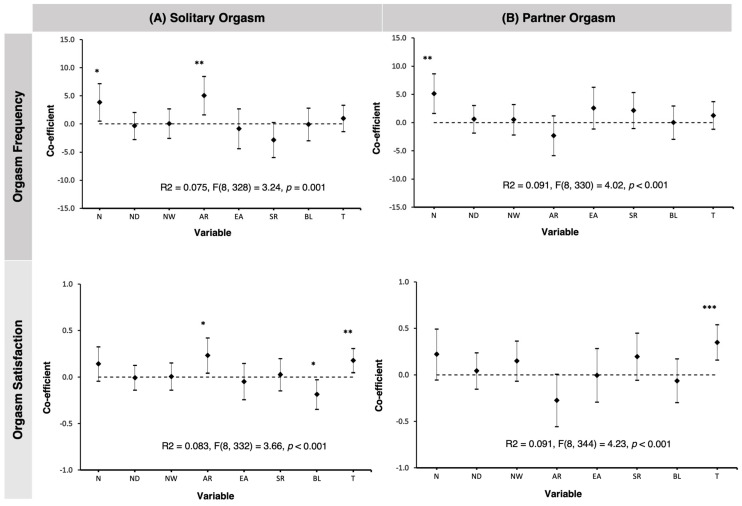
Multiple regression analyses. Dimensions of interoception as simultaneous predictors of orgasm frequency (**top row**) and satisfaction (**bottom row**). Panel (**A**) shows solitary orgasm frequency/satisfaction; Panel (**B**) shows partner orgasm frequency/satisfaction. Error bars indicate 95% confidence intervals. *** *p* < 0.001, ** *p* < 0.01, * *p* < 0.05. Note. The *Y*-axis shows dimensions of interoceptive awareness as indexed by subscales of the MAIA-2: N = Noticing, ND = Not Distracting, NW = Not Worrying, AR = Attention Regulation, EA = Emotional Awareness, SR = Self-Regulation, BL = Body Listening, T = Body Trusting.

Total MAIA scores were significantly positively correlated with both solitary orgasm frequency (r(316) = 0.17, *p* < 0.001) and partnered orgasm frequency (r(316) = 0.23, *p* < 0.001). We further examined how interoceptive awareness subscale scores predicted orgasm frequency (for both solitary and partnered interactions) via separate multiple regressions. They indicated that two MAIA subscales significantly predicted increased solitary orgasm frequency: Noticing (*B* = 3.86, *SE* = 1.69, *β* = 0.15, *t* = 2.28, *p* = 0.023, 95%CI [0.53, 7.18]) and Attention Regulation (*B* = 5.06, *SE* = 1.74, *β* = 0.21, *t* = 2.91, *p* = 0.004, 95%CI [1.64, 8.48])—see Figure 1 (Top Row, Panel A). For partner orgasm frequency, only the Noticing dimension was a significant positive predictor of frequency (*B* = 5.16, *SE* = 1.78, *β* = 0.19, *t* = 2.90, *p* = 0.004, 95%CI [1.66, 8.66])—see Figure 1 (Top Row, Panel B).

### 3.3. Orgasm Satisfaction (Solitary and Partner)

MAIA total scores were significantly correlated with both solitary orgasm satisfaction (r(316) = −0.19, *p* < 0.001) and partnered orgasm satisfaction (r(316) = −0.19, *p* < 0.001; lower satisfaction scores suggest higher satisfaction). Multiple regression analyses with the subscales of the MAIA showed that two significantly predicted increased solitary orgasm satisfaction: Attention Regulation (*B* = 0.23, *SE* = 0.10, *β* = 0.17, *t* = 2.40, *p* = 0.017, 95%CI [0.04, 0.42]) and Body Trusting (*B* = 0.18, *SE* = 0.07, *β* = 0.18, *t* = 2.70, *p* = 0.007, 95%CI [0.05, 0.31]), whereas Body Listening was significantly negatively associated with solitary orgasm satisfaction (*B* = −0.19, *SE* = 0.08, *β* = −0.17, *t* = −2.32, *p* = 0.021, 95%CI [−0.35, −0.03])—see Figure 1 (Bottom Row, Panel A). For partner orgasm satisfaction, only the Body Trusting dimension significantly predicted increased satisfaction (*B* = 0.35, *SE* = 0.10, *β* = 0.24, *t* = 3.61, *p* < 0.001, 95%CI [0.16, 0.54])—see Figure 1 (Bottom Row, Panel B).

## 4. Discussion

We hypothesised that self-reported interoceptive awareness would be positively associated with female orgasm frequency and satisfaction; findings vary depending on whether the sexual interactions are solitary or partner-based. Interestingly, we found that women rated their orgasm frequency and satisfaction significantly higher for solitary as compared to partnered sexual interactions. This finding is consistent with the evidence of the orgasm gap, which suggests that (1) women have lower orgasm rates for partnered sexual activities than men and (2) that this is especially true for women who have male partners, as compared to women who are bisexual or have female partners [30]. Our study indicates that the orgasm gap is not necessarily occurring because women cannot achieve orgasm, but rather, our findings demonstrate that orgasm is harder and less satisfying for women in partnered contexts.

When examining how trait-level interoceptive awareness relates to orgasm in women, we found that, as predicted, increased awareness was associated with both higher orgasm frequency and satisfaction. However, findings were more nuanced when considering the different facets of interoceptive awareness captured by the MAIA and how they related to orgasm frequency and satisfaction across solitary or partnered contexts.

The MAIA subscale of Noticing was a positive predictor of orgasm frequency for both solitary and partnered orgasms. In fact, it was the only interoceptive subscale to predict partnered orgasmic frequency. The Noticing subscale assesses one’s ability to distinguish one internal state from another and to notice specific sensations. The scale also includes an item that assesses one’s awareness of “tension” from within one’s body. Thus, when applied to a sexual context, the tendency to notice and accurately label internal states from sensations within the body may be reflected in more initiation and/or engagement with sexual interactions and behaviours. As a result, women with higher noticing tendencies may be better at detecting and distinguishing amongst subtle body cues, which could then translate into more frequent orgasms for both solitary and partnered sexual interactions. While previous literature has not yet directly investigated the relationship between interoception and orgasm frequency, our findings are theoretically in line with Handy and Meston’s [22] study, which found that women with higher MAIA Noticing scores had greater sexual concordance between their physiological and subjective genital arousal. These findings further underscore the importance of a woman’s ability to detect and self-report their sensory and sexual arousal sensations.

The MAIA subscale of Attention Regulation was also a significant positive predictor of solitary orgasm frequency and solitary orgasm satisfaction. Although not statistically significant, Attention Regulation was a negative predictor of partner orgasm frequency and satisfaction. Given that high scores on this subscale reflect an ability to sustain and control attention towards internal sensations, it may be that women who score higher on this trait are better able to maintain their arousal and enhance their sensory experiences (at least in solitary contexts), which could lead to a higher frequency of, and satisfaction with, orgasms. This could potentially be easier to achieve in solitary sexual activity since this allows for a woman to more easily concentrate their attention on specific bodily sensations without distraction. Partnered sexual interactions, on the other hand, involve additional emotional and social complexities that may make it more difficult to sustain attention to internal bodily sensations during sex. For example, attention may be split or ‘diluted’ if a woman feels simultaneously compelled to attend to both her own sensations and those of her partner. Theoretically, this may be linked to a common tendency to focus on one’s sexual performance from outside of one’s self, like a spectator, rather than focusing internally on their experience of the sexual interaction—termed “spectatorising” [31]—which may come at the cost of the interoceptive attention required to achieve (satisfying) orgasm in partnered contexts. Indeed, women who report more cognitive distraction during sexual activities report lower orgasm satisfaction [32].

Feminist theories suggest that spectatorising during sex is related to sexualisation/self-sexualised objectification in women [33], in addition to their negative influences on interoceptive awareness in women [34]. Sexualisation is an explicitly sexual form of objectification, which reduces one’s body into an “object” that is commodified by others and where one is denied elements of their personhood. Self-objectification/sexualisation occurs when one internalises such cultural messages, resulting in the objectification of oneself, reducing the self to a sexual object. Both self-objectification and sexualisation are associated with self-consciousness during sex and poorer sexual experiences [35]. Furthermore, Friedrickson and Roberts directly theorised that self-objectification negatively impacts one’s interoceptive awareness, leading to various psychological problems and sexual dysfunction in women [34]. Because self-objectification and spectatorising are a trend within the larger society, it is theoretically possible that female orgasmic satisfaction and frequency for partnered activities may be enhanced by encouraging women to focus and sustain their focus and attention on their own internal sensations during sex rather than focusing on their potential perceptions of their partner.

Our findings, as they pertain to the influence of MAIA Noticing and Attention regulation on orgasmic frequency in women, may provide mechanistic support that further explains why sensate-focused therapy, an evidence-based treatment used to treat sexual dysfunction in couples, appears to be effective [36]. Sensate-focused therapy encourages couples to initially focus on their internal sensations during non-sexual (but still sensual) touching of their bodies. Then, throughout treatment, touching becomes increasingly sexual, allowing couples to move away from the sexual “objectives” during sex and instead focus on noticing one’s own pleasurable bodily sensations arising during both sensual (non-sexual) and sexual touch partnered touch. Sensate-focused therapy has been previously criticised for lacking a mechanistic explanation [36]. However, our results suggest that it may exert beneficial effects by increasing aspects of interoceptive awareness (enhancing both noticing and attention regulation). Future research will be needed to test enhanced interoceptive awareness as a candidate mechanism underlying sensate therapy, ideally by including subjective measures of interoception pre- and post-treatment. More broadly, our findings suggest that encouraging women to “drop into their body” during partnered interactions to enhance interoceptive noticing and attention regulation capacities might increase orgasm frequency and satisfaction levels to match (or even surpass) those achieved during solitary contexts.

The MAIA subscale of Body Trusting predicted increased solitary orgasm satisfaction and was the only dimension of interoceptive awareness to also predict increased partner orgasm satisfaction. The Body Trusting subscale assesses one’s “experience of one’s body as safe and trustworthy.” Our results suggest women who feel at home/safe within their bodies and trust their body sensations are likely to report higher levels of orgasm satisfaction across solo and partnered contexts. The reverse also applies—women who have lower levels of Body Trusting are likely to report lower levels of orgasm satisfaction. One implication of this result is that enhancing Body Trusting could increase orgasm satisfaction for both solitary and partnered interactions, but the influence of Body Trusting may be particularly helpful for partnered sexual interactions since our effects were larger for the partnered domain. Future research could examine how interventions to increase ‘Body Trusting’ in particular facilitate orgasm in women. For example, previous research suggests that mindfulness training and yoga interventions can increase Body Trusting and underlying neural function [37,38]. However, whether similar training or other methods (such as cognitive behavioural therapy focused on body trusting) facilitate orgasm is an open question. Approaches to enhance body trusting in partnered sexual activities also warrant future research. For example, “bodily safety” could be enhanced during partnered sexual dynamics when a woman’s sexual partner models for her body trusting on their end. For example, this could potentially be conducted by respecting how the woman communicates about her body, its responsiveness, her bodily/sexual boundaries, and sexual desire. Coming from a place of respect and curiosity about the sexual responsiveness of one’s partner may help facilitate the women’s body trusting in comparison to implementing a mindset where the women’s partner(s) assume that they have a better understanding of the woman’s arousal and pleasure than the woman herself.

Interestingly, the Body Listening subscale of the MAIA was the only interoceptive dimension that predicted reduced solitary orgasm satisfaction in women. When engaging in Body Listening as defined by the MAIA, one’s attention may shift away from interoceptive sensations to related thoughts, behaviours, and feelings. Therefore, it may be that a general tendency to shift attention from the body and towards cognitions and behaviours has a detrimental impact on orgasm satisfaction, at least in solo contexts. For partnered contexts, this tendency may also exist but could be overridden by an overarching need for Body Trusting in order to obtain orgasm satisfaction. Future studies could examine whether other interoceptive tendencies might be present for partnered contexts when Body Trusting concerns are resolved.

Overall, our findings theoretically add an interoceptive component to Masters and Johnson’s human sexual response model [1]. For the excitement phase, our interoceptive findings suggest that noticing may play a significant role since noticing facilitates the detection of one’s sexual arousal and its initial changes. For the plateau phase, attention regulation appears to be critical since it maintains one’s focus on internal physiological sexual arousal signals and minimises cognitive distractions. Sustained attention towards one’s sexual arousal is required for the advancement towards the third orgasmic phase, a stage whereby vaginal and uterine muscles contract and a woman subjectively notices and reports having an orgasm prior to finishing with the resolution phase, where the body returns to being relaxed. Future studies should further examine how our interoceptive findings align with Masters and Johnson’s model of orgasm via causal modelling since our analyses cannot be used to make inferences about causal effects.

## 5. Limitations and Future Directions

Our findings include a few notable limitations. These include the fact that high numbers of our participants responded to our survey from feminist web forums, which could introduce bias into our study sample. For example, women within these forums may have different sexual attitudes and behaviours that contribute to their sexual tendencies than non-feminist-identifying women [39]. Future studies should replicate our study in samples that include non-feminist-identifying groups of women. Additionally, when looking at differences in the frequency and satisfaction of orgasms in solitary vs. partnered contexts, we did not account for looking at the participant’s relationship status, nor did we assess relationship satisfaction, which may specifically influence sexual functioning within partnered contexts. However, we used paired sample *t*-tests, which allowed us to examine differences in one’s own perception of their own solitary and partnered orgasms. Additionally, we did not directly examine the links between spectatorising, sexualisation/self-sexualised objectification, and interoceptive awareness; it would be of interest to further examine these theoretical links in future studies.

## 6. Conclusions

Research on healthy or optimal orgasmic functioning in women is sparse, with most research to date centred on orgasmic dysfunction. Our findings readdress this balance by identifying interoceptive awareness (and its specific dimensions) as an individual difference trait that predicts orgasm frequency and satisfaction across solitary and partnered contexts. Our findings also appear to have identified interoceptive-specific factors that are associated with an interoceptive model of orgasm frequency for women. Aligning our results with the Masters and Johnson model may contribute to the improved assessment and interventions that enhance normal orgasmic functioning in women. It may also assist in the identification of specific treatment mechanisms (i.e., interoceptive noticing, attention regulation, and body trusting) that further explain why pre-existing treatments such as Sensate Focused Therapy may be helpful for improving sexual functioning in couples. Thus, our results pertaining to partnered interactions may lead to increasing the pleasurable sexual encounters for women in partnered sexual interactions and, thus, potentially contributing to a reduction in the orgasm gap. Ultimately, the implications of our findings may lead to the enhancement of orgasmic frequency and satisfaction in women, which may contribute to improved female wellbeing and relationship satisfaction and, thus, benefit the wider society.

## Figures and Tables

**Table 1:** Demographics.

Variable	N	%	*M*	*SD*	Min	Max
Age	360		29.56	10.29	18	69
Gender Identity						
Female	341	94.7				
Male	2	0.6				
Non-Binary	17	4.7				
Sexual Orientation						
0 (Heterosexual)	79	21.9				
1	75	20.8				
2	73	20.3				
3	80	22.2				
4	21	5.8				
5	17	4.7				
6 (Homosexual)	8	2.2				
No Sexual Attraction	7	1.9				

**Table 2:** Comparison between original FOS questions and adapted FOS-Solitary questions.

FOS-Partner (Original Questions)	FOS-Solitary (Adapted Questions)
1. How often do you have an orgasm from vaginal penetration only (no direct clitoral stimulation) during intercourse with a partner?	1. How often do you have an orgasm from vaginal penetration only (no direct clitoral stimulation) during solitary masturbation?
2. How often do you have an orgasm from intercourse with a partner that includes both vaginal penetration and clitoral stimulation?	2. How often do you have an orgasm from solitary masturbation that includes both vaginal penetration and direct clitoral stimulation?
3. How often do you have an orgasm from HAND/MANUEL stimulation of your genitals/clitoris by a partner?	3. How often do you have an orgasm from HAND/MANUEL stimulation of your genitals/clitoris by yourself?
4. How often do you have an orgasm when you yourself manipulate or rub your own genitals/clitoris when you are with a partner?	4. How often do you have an orgasm when you yourself manipulate or rub your own genitals/clitoris by yourself?
5. How often do you have an orgasm from ORAL stimulation of your genitals/clitoris by a partner?	5. How often do you have an orgasm from stimulation of your genitals/clitoris with sex toys during solitary masturbation?
6. In general, how satisfied/unsatisfied are you with the number of orgasms that you have during sexual activity with a partner?	6. In general, how satisfied/unsatisfied are you with the number of orgasms that you have during solitary masturbation?
7. In general, how satisfied/unsatisfied are you with the quality or experience of orgasms that you have during sexual activity with a partner?	7. In general, how satisfied/unsatisfied are you with the quality or experience of orgasms that you have during solitary masturbation?

**Table 3:** Pearson’s r correlations between age, sexual orientation, and various study variables.

Variable	Age	Sexual Orientation
Interoceprion	0.028	0.056
Sig. (2-tailed)	0.601	0.293
N	360	353
Solitary orgasm frequency (%)	−0.066	0.087
Sig. (2-tailed)	0.229	0.12
N	329	332
Solitary orgasm satisfaction	0.071	−0.021
Sig. (2-tailed)	0.194	0.712
N	333	326
Partner ogasm frequeny (%)	0.023	0.059
Sig. (2-tailed)	0.682	0.289
N	331	326
Partner orgasm satisfaction	0.1	−0.086
Sig. (2-tailed)	0.063	0.113
N	345	338

**Table 4:** Means, standard deviations, and correlations between key study variables.

	M	SD	(1)	(1.1)	(1.2)	(1.3)	(1.4)	(1.5)	(1.6)	(1.7)	(1.8)	(2)	(3)	(4)
(1) Interoception	2.7	0.62	-											
(1.1) Noticing	3.52	0.96	0.58 **	-										
(1.2) Not Distracting	1.83	1.13	0.29 **	−0.02	-									
(1.3) Not Worrying	2.39	1.06	0.26 **	−0.06	−0.13 *	-								
(1.4) Attention Regulation	2.62	1	0.77 **	0.45 **	−0.02	0.15 **	-							
(1.5) Emotional Awareness	3.66	0.99	0.66 **	0.53 **	−0.02	−0.06	0.48 **	-						
(1.6) Self-Regulation	2.62	1.14	0.70 **	0.30 **	−0.04	0.12 *	0.49 **	.48 **	-					
(1.7) Body Listening	2.42	1.27	0.70 **	0.41 **	0.07	−0.08	0.47 **	0.55 **	0.58 **	-				
(1.8) Body Trusting	2.88	1.4	0.67 **	0.26 **	0.19 **	0.18 **	0.43 **	.22 **	0.46 **	0.45 **	-			
(2) Solitary orgasm frequency (%)	65.46	24.56	0.17 **	0.21 **	−0.01	0.02	0.21 **	0.10	0.03	0.10	0.11 *	-		
(3) Solitary orgasm satisfaction	6.04	1.33	0.19 **	0.12 *	0.02	0.08	0.21 **	0.06	0.10	0.02	0.22 **	0.37 **	-	
(4) Partner orgasm frequency (%)	44.49	25.55	0.23 **	0.25 **	0.03	0.02	0.12 *	0.21 **	0.19 **	0.17 **	0.15 **	0.37 **	0.15 **	-
(5) Partner orgasm satisfaction	4.88	2.07	0.19 **	0.11 *	0.03	0.11	0.07	0.07	0.18 **	0.11	0.27 **	−0.03	0.18 **	0.59 **

** Correlation is significant at the 0.001 level (2-tailed); * Correlation is significant at the 0.01 level (2-tailed); Listwise N = 318.

## Data Availability

The original contributions presented in this study are included in the article. Further inquiries can be directed to the corresponding author.
